# A device-specific prioritization strategy based on the potential for harm to human health in informal WEEE recycling

**DOI:** 10.1007/s11356-017-0390-7

**Published:** 2017-10-20

**Authors:** Alessandra Cesaro, Vincenzo Belgiorno, Mentore Vaccari, Aleksander Jandric, Tran Duc Chung, Maria Isabel Dias, Andrew Hursthouse, Stefan Salhofer

**Affiliations:** 1 Sanitary Environmental Engineering Division (SEED), Department of Civil Engineering, University of Salerno, Via Giovanni Paolo II, 84084 Fisciano, SA Italy; 2 University of Brescia, Via Branze 43, 25123 Brescia, Italy; 3 Waste Management Institute, BOKU University, Muthgasse 107, 1190 Vienna, Austria; 4 Instituto Superior Tecnico, Campus Tecnologico e Nuclear, Universidade de Lisboa, Estrada Nacional 10, Bobadela, 2695-066 Loures, Portugal; 5 University of West Scotland, Paisley Campus, Paisley, PA1 2BE UK

**Keywords:** Electronic waste, Hazard, Metals, Sanitary environmental risk, Toxicity

## Abstract

In developing countries, the recovery of valuable materials from Waste Electrical and Electronic Equipment (WEEE) is carried out via uncontrolled practices, posing potentially severe risks both to human health and the environment. The assessment of the risk, which depends on both the kind and hazardous properties of the substances contained in WEEE, is currently limited as the exposure scenario for the single informal practice cannot be fully characterized for this purpose. In this context, this work proposes and evaluates a strategy to identify the relative potential harm of different kinds of WEEE by their content in metals, selected as the target substances of concern. This was based on the individual metal content, primarily located in the printed circuit boards (PCBs) of the different devices. The metal composition of the individual PCBs was identified and the dominant unregulated metal recovery practices were reviewed to identify the most suitable parameter to express the toxicity of these metals. Based on a mass-normalized cumulative toxicity, via the inhalation route, individual components were assessed from compositional variation found in the literature. The results is a semiquantitative ranking of individual components, revealing significant differences in potential harm posed by different electronic appliances and an opportunity to provide prioritization strategies in future management.

## Introduction

The rapid innovation in digital technology in the last century has resulted in a dramatic increase in the production of Waste Electrical and Electronic Equipment (WEEE) (Ongondo et al. 2011; Kiddee et al. 2013). Its generation was estimated to be 41.8 million tonnes in 2014 and it is expected to increase to 65.4 million tonnes by 2017 (Breivik et al. 2014). WEEE includes several categories of end-of-life electrical appliances, so that it is a highly heterogeneous waste flow (Cucchiella et al. 2015; Golev et al. 2016). However, the main material constituent is the metallic fraction, accounting for approximately 65% of the total weight of electric and electronic equipment and including base and precious metals (Jaiswal et al. 2015). Due to the presence of valuable metals, WEEE is now regarded as urban stock, available for the mining of both precious metals and rare earth elements (REEs). The latter have received a great deal of recent attention as their supply is sensitive to many factors: REEs are provided predominantly from China and export has been limited, posing an issue of supply for conventional industrial applications (Dutta et al. 2016). The possible recovery of these strategic materials along with other valuable metals from WEEE is an important driver for the implementation of WEEE recycling practices (Binnemans et al. 2013; Tunsu et al. 2015).

In developed countries, the recovery of materials from waste flows is also a legal obligation (Li et al. 2013; Favot et al. 2016; Morris and Metternicht 2016; Zhou et al. 2017) with the procedures for the operation of recycling processes formally identified and regulated, in order to reduce environmental impact. Conversely, in developing countries, informal recycling methods are very diverse (Ardi and Leisten 2016; Salhofer et al. 2016): mechanical processes, open burning, and chemical leaching are applied under uncontrolled conditions, with the aim of liberating the components of interest from the discharged electronic appliances. However toxic substances are also released into the environment and, due to the absence of emission control systems, they can pose severe risks to both human and environmental health (Tsydenova and Bengtsson 2011; Long et al. 2013; Cao et al. 2016).

WEEE can contain a range of hazardous substances, which include potentially toxic elements (e.g., mercury, cadmium, lead) and flame retardants (e.g., pentabromophenol, polybrominated diphenyl ethers (PBDEs), tetrabromobisphenol A (TBBPA)) (Tsydenova and Bengtsson 2011).

Once released into the environment, hazardous substances can negatively affect human health through different exposure routes (Leung et al. 2008; Sepúlveda et al. 2010; Tang et al. 2010; Wei and Liu 2012; Song and Li 2015; Zeng et al. 2016), particularly the workforce or the population living in the neighborhood of informal recovery sites (Chan and Wong 2013; Sepúlveda et al. 2010). Workers suffer negative health effects by exposure through skin contact and inhalation, while the wider community is exposed to the contaminants through smoke, dust, drinking water, and food contamination (Robinson 2009).

Risk assessment is the evaluation of the potential adverse health effects on humans exposed to environmental hazards. It is carried out through the following steps (Zhang et al. 2010): (i) the identification of the potential hazards associated to the presence of selected contaminants into the environment; (ii) assessment of the exposure conditions (i.e., intensity, frequency, and duration of the exposure); (iii) assessment of the contaminant toxicity; and (iv) characterization of the risk, as the probability that the identified contamination phenomena can produce the loss of human life. Under the framework of risk assessment in informal WEEE recycling, the detailed process applied play a key role (Grant et al. 2013), influencing the mobility of hazardous substances and the extent of the environmental contamination. Shredding practices produce mainly dust that can contain both flame retardants (Morf et al. 2005) and heavy metals (Song et al. 2015). Open burning generates smokes with a variety of organic pollutants and heavy metals (Awasthi et al. 2016), whose presence is tightly related to the operating thermal conditions: reductive atmosphere promotes the evaporation of heavy metals like cadmium and zinc at lower temperatures (Dong et al. 2015; Yu et al. 2016). Moreover, the uncontrolled combustion of plastics containing brominated flame retardants has been largely reported to promote the formation of polybrominated dibenzo-p-dioxins/dibenzofurans (Tue et al. 2016).

Regardless of the specific informal treatment process (i.e., shredding, open burning), it is reasonable to assume that the relative risk for the exposed community, either workers or population, will be strongly related to the type of device being processed, and the variation in composition in terms of hazardous substance content. It indeed determines the presence and amount of hazardous substances available for potential release to the environment.

Toxic metals have been recognized as substances of particular concern (Tsydenova and Bengtsson 2011) and they are concentrated in specific WEEE components, such as printed circuits boards (PCBs), which are present in a wide variety of electric and electronic appliances (Oguchi et al. 2011). The hazard from different types of WEEE is mainly related to the total mass of metals contained in the PCB of each appliance as well as to the intrinsic toxicity of the metal itself.

Although the issue of the risks posed by the informal recycling of WEEE has been debated in the literature (Zhang et al. 2010; Tsydenova and Bengtsson 2011), the potential harm to human health from discharged electric and electronic devices has yet to be quantified. This work proposes and evaluates a methodology to categorize different WEEE by their relative potential for harm, assessed by reference to the metal content of their PCBs. In order to identify the most suitable parameter to express the metal toxicity, data on the possible routes for the release of these metals into the environment are discussed with reference to the more commonly reported informal recycling practice.

## Methodology

The approach was to investigate and evaluate a strategy to test the significance of the metal content to define the harmful potentiality of different types of WEEE during informal recycling practices.

This was based on the metal composition of different end-of-life appliances, derived from previously published assessments. As highlighted above, data focus on the metal content of printed circuit boards (PCBs) where the majority of metals are present and are widely used in electric and electronic appliances (Oguchi et al. 2011). Also, the extensive compositional analysis of the metal content of PCBs ensures that there is an opportunity to consider a wide range of potentially harmful elements and the comparative assessment of WEEE constituents are more representative of likely exposure/risk during informal recycling.

It is worth pointing out that substances of concern other than metals (i.e., flame retardants) could not be considered due to the lack of data on their content in different electric and electronic appliances.

### The composition of PCBs in terms of metal content

The material composition of PCBs is a complex and much debated subject with high economic potential on the one side and the presence of hazardous components on the other. PCBs differ in size, function, and material composition and they should be perceived as a method for construction of an electronic circuit, rather than a distinctive electronic component.

Even though the literature presents numerous studies of the material composition of PCBs, their relevance and comparability is limited. The reasons for this are:

- insufficient information on the type of PCB that is analyzed as well as the year of production of the electronic device it belongs to: PCBs from personal computers vary in size and material composition, such as motherboard, RAM, or power supply PCBs;
- many of the PCB metals are in the milligram per kilogram range and the results of the chemical analyses are highly dependent on the method applied to assess their concentration;
- material composition data often represents composite results of repeated experiments with statistical significance or methods missing.

Consequently, the data used in our study were selected on the basis of both the background information on the PCBs analyzed and the extent of electronic categories investigated, as given in Table 1.

**Table 1:** Material composition of PCBs from different electronic devices, expressed as milligram per kilogram

PCB type	Base metals							Precious metals			Other metals									Reference
	Cu	Fe	Al	Pb	Sn	Zn	Ni	Pd	Au	Ag	Ba	Be	Bi	Cd	Cr	Co	Ga	Sb	Ta	
Calculator	30,000	40,000	50,000	–	–	–	–	5	50	260	–	–	–	–	–	–	–	–	–	Hagelücken and Corti (2016)
DVD player	135,000	315,500	37,000	12,000	22,000	26,000	–	12	83	413	4300	–	85	2	320	110	9	1200	77	Hagelücken and Corti (2016); Oguchi et al. (2011); Oguchi et al. (2013)
Gas discharge lamps	5389	5879	4214	1470	1323	686	54	1	1	11	–	–	–	–	10	–	–	–	–	Huisman et al. (2008)
Mobile phone	423,875	16,325	13,300	12,163	36,925	4825	10,533	137	1067	2171	19,000	11	220	2	105	140	70	880	1300	Camelino et al. (2015); Oguchi et al. (2011); Oguchi et al. (2013); Cucchiella et al. (2016)
PC	196,000	23,860	22,400	17,760	20,600	9866	1433	95	428	875	3480	8	83	3	400	58	19	1625	1422	Oguchi et al. (2011); Oguchi et al. (2013); Cucchiella et al. (2016)
Monitor	100,000	300,000	15,000	–	–	–	–	10	20	280	–	–	–	–	–	–	–	–	–	Hagelücken and Corti (2016)
Portable audio	210,000	230,000	10,000	–	–	–	–	4	10	150	–	–	–	–	–	–	–	–	–	Hagelücken and Corti (2016)
Power tools	160,000	41,000	58,000	30,000	27,000	14,000	1100	48	18	1100	–	–	–	–	210	–	–	–	–	Huisman et al. (2008)
Printer	166,000	26,500	125,500	5500	18,150	5750	54,000	21	54	40	3000	–	9	–	32	220	3	530	–	Yoo et al. 2009; Oguchi et al. (2011); Oguchi et al. (2013)
TV (CRT, PDP, LCD)	173,400	30,420	47,980	10,720	20,000	18,260	6750	10	105	1848	2825	–	127	6	52	18	–	2200	50	Williams (2010); Oguchi et al. (2011); Oguchi et al. (2013); Cucchiella et al. (2016)
Cassette recorder	150,000	48,000	48,000	18,500	21,000	13,500	–	42	25	190	1300	–	230	7	140	28	11	2150	16	Oguchi et al. (2011); Oguchi et al. (2013)
Camera	250,000	35,000	25,667	21,333	38,667	10,267	–	390	770	3733	16,667	10	157	1	1933	107	22	1967	5300	Oguchi et al. (2011); Oguchi et al. (2013); Cucchiella et al. (2016)
Portable CD/MD player	265,000	45,500	47,500	10,650	49,000	15,500	–	280	655	3550	13,800	60	880	–	2385	115	–	1300	5135	Oguchi et al. (2011); Oguchi et al. (2013)
Game console	190,000	77,000	40,000	13,000	26,000	12,000	–	43	230	740	5100	–	260	1	800	100	16	2900	83	Oguchi et al. (2011); Oguchi et al. (2013)

### Informal recycling methods for PCBs and exposure routes

Both direct and indirect exposure pathways to the substances released from informal WEEE recycling have been studied (Frazzoli et al. 2010; Heacock et al. 2015). They are often related to specific informal recycling practices (Huo et al. 2007; Asante et al. 2012), which are recognized to be differently applied in diverse world regions. Large organized informal communities are present in China and India, while in Africa those activities are carried out by individuals (Schluep et al. 2009).

In China, the most dominant areas for informal treatment activities are Guiyu, in Guandong Province, and Taizhou, in Zhejiang, where the processing of PCBs focuses on the recovery of metals, especially gold, while the nonmetallic materials are landfilled (Brigden et al. 2005; Guanghan et al. 2016). The components with the highest gold content, namely silicon chips and contacts, are thus removed from PCBs and treated by leaching with acids, such as nitric and hydrochloric acids (Wang et al. 2013), for gold recovery (Wen et al. 2006; Schluep et al. 2009). The rest of the circuit boards often goes to an acid recovery of the remaining metals (Schluep et al. 2009), but open burning has been reported as another method to treat the rest of the PCBs (Wang et al. 2013). The Chinese informal sector appears thus to rely on a number of different recycling methods: physical dismantling, heat-assisted removing of components from PCBs, chipping plastics, and melting as well as open burning for either recovery or disposal purposes are highlighted in particular (Chi et al. 2011).

A similar variety of informal recycling practices has been observed in India, where WEEE recycling takes place through traders, dismantlers, and recyclers. In Bangalore, identified as the country’s information technology hub (Liu et al. 2016), the preprocessing of broken equipment includes dismantling and sorting of the waste stream into several groups: CRT, plastics, PCBs, wires and cables, and metals (Keller 2006). PCBs are dismantled into boards without electronic components, connectors, and copper. To de-solder PCBs and to recover gold, different techniques are applied. Solders are melted by using heat from an open-frame kerosene burner (Brigden et al. 2005) or coal-fire grills. Silicon chips are removed from circuit boards by putting them in a heated pool of molten lead-tin solder, and later, processed for gold extraction by using acid baths (Keller 2006; Rochat et al. 2007; Schluep et al. 2009). The rest of the boards are burned at large-scale burning facilities or leached in acid to partially recover remaining metals (Schluep et al. 2009). The residual, non-valuable fractions from those steps normally end up in open dump sites.

Different information is available for activities in Africa, where the most prominent country for informal e-waste processing is Ghana. The absence of legislation clearly banning the import of both WEEE and UEEE (Used Electric and Electronic Equipment) (Li et al. 2013), makes indeed Ghana as an eligible destination country for the illegal import of WEEE that, in turn, feeds the informal recycling sector.

In Ghana the most common practices are the manual dismantling to salvage copper and other metal-rich parts for resale (Huang et al. 2014). Dismantled components, cables, and wires are burned to extract copper (Amoyaw-Osei 2011; Huang et al. 2014). The unusable fractions from dismantling, such as plastics, are accumulated and regularly burned to reduce volume or dumped without further treatment (Amoyaw-Osei 2011). Chemical leaching processes for precious metal recovery from PCBs have not been observed in African countries (Schluep et al. 2009). In the case of Ghana, PCBs are ground into fine powder and exported to Asian countries, mainly China and India (Grant and Oteng-Ababio 2012).

The evidence of the adverse impacts on the environment and human health from these crude methods have been largely discussed in the literature. Several studies (Brigden et al. 2005; Leung et al. 2008; Sepúlveda et al. 2010; Tang et al. 2010; Amoyaw-Osei 2011; Wei and Liu 2012) have identified the high concentration of both metals (such as lead, nickel, copper, cadmium), and organic pollutants in dust, sediment, and wastewater from recycling workshops or in soil and water from open pools close to recycling facilities in different regions worldwide.

Although not a comprehensive study, the practices reported to be applied in these areas could be considered as representative of the informal recycling activities, which include manual dismantling, size reduction, open burning, and acid leaching (Sepúlveda et al. 2010). Each of these uncontrolled processes affects the environmental quality through different routes (Tsydenova and Bengtsson 2011) and, in turn, human health. However, persistently poor ventilation of dusty working areas, poor hygiene, the absence of or improper use of both personal protective equipment (such as respirators), and emission control systems increase the likelihood of significant exposures mainly through inhalation, and aggravate the risk from lung related diseases (Rim et al. 2013).

### Approach to the assessment of the potential for harm to human health from PCBs

Risk analysis is a useful tool to quantify the probability that the application of particular informal practice can lead to the loss of human life, providing technical data to describe the hazard that the practice itself may entail. However, the relative characterization of the risk from different informal practices seems to be limited by the lack of data on the contaminants emitted, so that it is not possible to identify the most hazardous activity. As these practices are carried out under uncontrolled operating conditions, it is indeed hard to define the chemical form and the physical state of the released contaminant, as discussed for different heavy metals in the study of Dinis and Fíuza (2011).

It is worth noting that the variability in WEEE composition can also influence the extent of the risk, as the release of hazardous substances into the environment depends on their presence and availability in different devices. In turn, the potential harm to human health from hazardous substances is related to their toxicological characteristics.

Due to the severe uncertainties in figuring out the exposure scenario for relative risk assessment, this work aims at proposing and evaluating a methodology to classify different types of WEEE by their relative potential hazard, which is estimated taking into account both the concentration and the toxicological properties of hazardous substances, namely metals, in their PCBs.

Published data on the categorization of different types of WEEE have previously been based on both the concentration and the total amount of toxic metals in their PCBs. Oguchi et al. (2013) points out that mobile phones and other small digital items such as portable audio players and digital cameras have high to moderate concentrations as well as moderate total mass of toxic metals, like chromium, barium, and lead in comparison to bigger appliances. For this reason, they were recognized as high priority items, when managing toxic metals in WEEE. On the other hand, the total amounts of toxic metals contained in other midsized items such as audio/video devices and ICT equipment, including printers, were not negligible, but their concentration was not particularly high (Oguchi et al. 2013). However, this assessment focused only on the quantity of a few selected metals.

For the present work, standardized database from environmental risk assessment was used (US-EPA 2016).

The impact of environmental exposure determines the risk assessment of potentially toxic elements. Based on the study of informal recycling methods, the toxicity inhalation path was considered as the most relevant and the corresponding toxicity value, namely the inhalation reference concentration (RfC), was selected for each metal (Table 2). The RfC is an estimate of a concentration under continuous exposure for individuals that does not present any risk of deleterious effects during a lifetime. Selected RfC values referred to the elemental metal or, if not available, to a metal compound that is likely to be produced during informal recycling practices, such as open burning.

**Table 2:** Reference concentrations selected as toxicity values (US-EPA)

Metal	RfC [μg/m^3^]
Aluminum (Al)	5
Barium (Ba)	0.5
Beryllium (Be)	0.02
Cadmium (Cd)	0.01
Chromium (Cr)	0.1
Cobalt (Co)	0.006
Lead (Pb)	0.2
Nickel (Ni)	0.014
Strontium (Sr)	0.2

For the identified PCBs, the contribution to the potential for harm indicator of the i-th metal (PHI_i_) was calculated as the ratio between its concentration in the PCB and the correspondent RfC. The total indicator of the potential for harm (PHI), based on the presence of the “n” contaminants, was then assessed through the following expression:

$$ PHI=\sum_{\mathrm{i}=1}^{\mathrm{n}}{\mathrm{PHI}}_{\mathrm{i}} $$

**Fig. 1:**
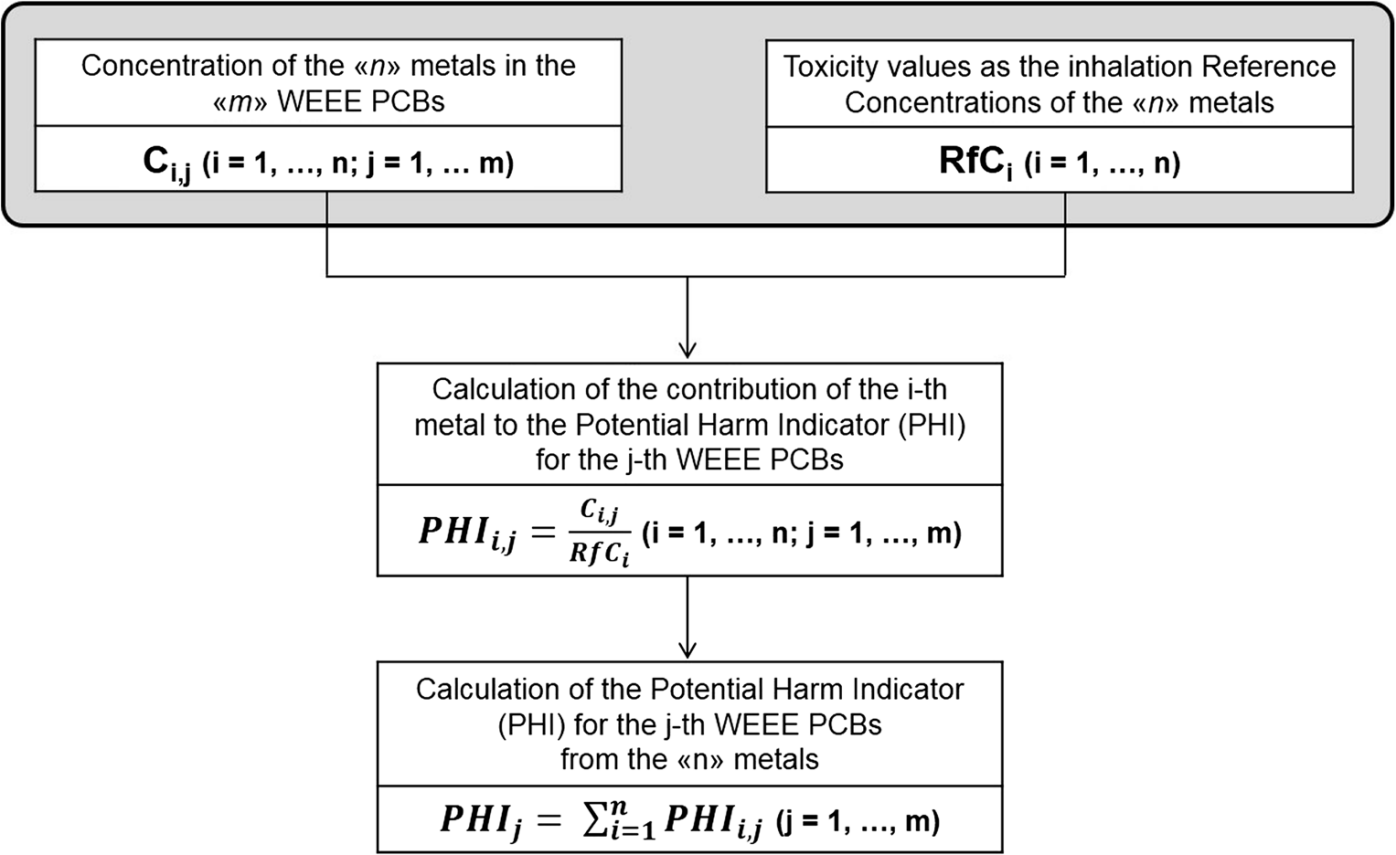
Flow chart of the developed methodology

A schematic of the construction of the indicator is shown in Figure 1.

The comparative analysis of the PHI of PCB was also referred to a normalized PHI (DPHI), which was calculated as the ratio between the PHI of the single PCB and the minor PHI.

## Results and discussion

The methodology provides a simple potential for harm indicator (PHI), expressed as an inverse reference concentration referred to the mass of the PCB rather than the metal. This indicator highlights the significance of specific WEEE components relative to each other. The higher the value of PHI, the more significant hazard a particular WEEE component may be for human health. The weight of individual appliances does not play any role in the definition of the PHI, as the results are normalized per mass unit of the device to allow a suitable comparison between different sizes of WEEE. The relevance of this work is in its use to supply a means of classification of components, which may provide a role in prioritization of decision making in management of waste streams, as highlighted in Table 3.

**Table 3:** Relative potential harm of selected WEEE

PCB type	DPHI	DPHI_no Al_
Printer	1.977	347
Mobile phone	445	78
TV (CRT, PDP, LCD)	278	48
Power tools	121	20
PC	109	19
Camera	92	16
Portable CD/MD player	69	11
Cassette recorder	56	9
Game console	55	9
DVD player	50	8
Gas discharge lamps	6	1
Calculator	5	–
Monitor	2	–
Portable audio	1	–

According to these results, the significance of the PCBs from particular WEEE types is:

- printer > mobile phone > TV > power tools > PC > camera > portable CD/MD player > cassette recorder > game console > DVD player > gas discharge lamps > calculator > monitor > portable audio.

Therefore, when considering the sustainable management of WEEE, printers should be considered at the highest level of priority. The PHI for printers was found to be approximately 2000 times higher than that of the portable audio, which is the lowest. According to the order of magnitude of the DPHI, the other PCB types can be clustered in the following classes:

- class 1, including mobile phone, TV, power tools, and PC, with PHI values from 445 to 109 times that for portable audio;
- class 2, consisting of camera, portable CD/MD player, cassette recorder, game console, and DVD player, whose PHI values were in the range 50–92 times higher than that of portable audio;
- class 3, composed of gas discharge lamps, calculator, monitor, and portable audio, with DPHI lower than 10.

With the exception of the game console and gas discharge lamps, belonging to the categories n. 7 (toys, leisure, and sport equipment) and n. 5 (lighting equipment) of the European WEEE Directive respectively; the considered devices are listed in either the category n. 3 (IT and telecommunications equipment) or the category n. 4 (consumer equipment) of the same Directive.

As pointed out by (Tansel 2017), the quantities of discarded electronic consumer products have increased exponentially, due to advancing technology, manufacturing processes, rapid market penetration, and planned obsolescence. However, for a large portion of this waste, recycling is not properly documented, suggesting it is likely to be handled under uncontrolled conditions, with consequences for risks to both human and environmental health. Further efforts should be made to provide a barrier to exposure and the categorization of WEEE by their PHI indicates the order of priority that should be followed in defining the strategies for the traceability of different kinds of WEEE. This may allow the adoption of basic, easy-to-apply practices during the informal recycling of the appliances.

The methodology also highlights that the individual content of metals is not sufficient for prioritization of WEEE management.

This work highlights printers as the most significant component of WEEE, with high content in aluminum, nickel, and cobalt. The less harmful category (portable audio) has typically lower concentrations of aluminum and nickel as well as cobalt being absent.

Although such outcomes seem to suggest a linear relationship between the concentration of these metals and the PHI value, the results obtained for the other devices do not support this conclusion, as the potential danger from a specific device is related directly to the toxicity potential of its constituents. In the studied PCBs, metals like cobalt are present at low concentrations, but the corresponding reference concentration is also very low, indicating a high toxic potential. Conversely, aluminum is one of the main constituents of PCBs, but its toxicity expressed as reference concentration is three order of magnitude greater than that of cobalt.

The analysis of the ranking results, shown in Table 3, identifies that the aluminum concentration (13,300 mg/kg) of mobile phone PCB cannot be related to the corresponding PHI value, as observed for printers.

Although the concentrations of aluminum are very high, ranging between 4214 and 125,500 mg/kg, the presence of this substance do not affect considerably the potential for harm of the considered PCBs: in fact, due to the low toxicity of this metal, the priority ranking based on the PHI values do not change if not considering the presence of aluminum, as shown in Table 3 (DPHI_no Al_). Different consideration raise for nickel, whose presence drives the definition of the PHI values for the PCB of the WEEE types clustered in class 1. Although most of these devices contain cobalt, which is even more toxic than nickel, the latter is present in concentrations approximately 100,000-fold higher than the corresponding RfC. Similarly, for the devices grouped in class 2, lead is the metal characterized by a concentration ranging between 12,000 and 21,300 mg/kg, which is up to 100,000-fold higher than its RfC. The contribution of other metals, like barium, cadmium, and chromium, to the overall PHI determines the order of priority of the single WEEE type PCBs within each cluster, namely class 1, class 2, and class 3. This analysis suggests that, when the metal concentration is, at least, 50,000-fold higher than the RfC, its presence drives the definition of the potential harm of the corresponding PCB.

It is worth identifying that all appliances contain large amounts of copper and iron and most of them also contain other metals like zinc that do not contribute to the assessment due to the lack of comparable toxicity data. It is therefore important that data should be generated to refine the model and subsequent classification of WEEE components.

In the wider context of environmental risk assessment, the absence of inhalation route data on a number of elements limits the evaluation of the risk to individuals exposed to either dust or gaseous emissions from informal WEEE recycling practices. Although the concentration in air of some metals, including copper and iron, has been reported in working places where either dismantling or other uncontrolled recycling practices are performed (Julander et al. 2014; Zeng et al. 2015), it is still not possible to verify the effects of those concentrations to human health after a chronic exposure. Similarly, the identification of correlation between health effects and metal concentrations (Perkins et al. 2014) do not provide suitable information to address the definition of risk-based procedures.

This methodology represents a possible approach to address this gap and needs to be widened with reference to both components of WEEE and individual substance toxicity. Field studies focused on the monitoring of substances released during informal WEEE treatment would further promote the verification of exposure conditions for either recyclers or population living in the surroundings of working sites.

The prioritization of control measures in the sustainable management of WEEE needs to take into account the device as well as the PCB. Further refinement can be made by identifying metal speciation and toxicity of specific compounds likely to be encountered during the processing of the waste. In addition, other toxic substances should also be considered as their adverse effects on both environment and human health have been extensively reported (Herat and Agamuthu 2012). To this end, further efforts should be directed towards the quantification of nonmetallic substance of concern in electric and electronic appliances.

## Conclusions

This work proposes a methodology to assess the relative potential for harm to human health from the informal recycling of different types of WEEE. The informal processing of WEEE, which is largely performed in developing countries, poses a severe risk for both the human health and the environment, related to the possible release of toxic substances during the uncontrolled treatment of waste components. Rudimentary shredding and open burning are among the most commonly reported procedures applied to recover valuable materials, and they raise great concern due to the potential for inhalation of contaminated air by either workers or people living in the surrounding of the informal working sites.

This methodology was able to provide the potential harm indicator (PHI), which takes into account both the amount and the toxicological properties of the metals of concern, primarily present in the printed circuit boards. The total quantity as well as the toxicological properties of these metals is the main factor contributing to the overall potential for harm of discharged electronic devices. The potential harm from different types of WEEE can be driven by the presence of the more toxic metals that are of a significant mass. However, when the content of these metals is lowered, the potential harm is driven by the relative content of the toxic elements.

Printers were identified as the most hazardous type of WEEE, followed by several kinds of both IT and consumer appliance, which should be regarded as high-priority devices when considering their informal treatment.

This methodology represents a useful tool for WEEE management, indicating an order of priority for the definition of both strategies and easy-to-apply practices aimed at reducing the extent of adverse effects during the informal processing of the appliances.

However, there is an urgent need for further studies, looking at a more comprehensive characterization of the hazardous substances in different types of WEEE components. Data identification and collection should be undertaken along with field studies to validate the results from the assessment. An understanding of the specific informal recycling methodology is also of interest as it will then identify most appropriate exposure models.
